# A New Dinuclear Cobalt Complex for Copolymerization of CO_2_ and Propylene Oxide: High Activity and Selectivity

**DOI:** 10.3390/molecules25184095

**Published:** 2020-09-08

**Authors:** Wen-Zhen Wang, Kai-Yue Zhang, Xin-Gang Jia, Li Wang, Lei-Lei Li, Wei Fan, Li Xia

**Affiliations:** College of Chemistry and Chemical Engineering, Xi’an Shiyou University, Xi’an 710065, China; kyzhang000@163.com (K.-Y.Z.); jiaxingang76@xsyu.edu.cn (X.-G.J.); lwang2018@xsyu.edu.cn (L.W.); lll@xsyu.edu.cn (L.-L.L.); vfan111@163.com (W.F.); lxia9264@163.com (L.X.)

**Keywords:** carbon dioxide, propylene oxide, poly(propylene carbonate), copolymerization

## Abstract

Based on the ligand H_4_Salen-8*^t^*Bu (salen-**4**), a new dinuclear cobalt complex (salen-**4**)[Co(III)TFA]_2_ (salen-**4** = 3,5-di-*tert*-butylsalicylaldehyde-3,3′-diaminobiphenylamine; TFA = trifluoroacetic acid) has been firstly synthesized and characterized. It shows high catalytic activity for the copolymerization of propylene oxide (PO) and carbon dioxide (CO_2_), yielding regioregular poly(propylene carbonate) (PPC) with little generation of propylene carbonate (PC) by-product. It has been found that (salen-**4**)[Co(III)TFA]_2_ shows higher activity at milder conditions, generating a polymer with maximum Mn of 293 kg/mol and a narrow molecular weight distribution PDI of 1.35. The influences of reaction time, CO_2_ pressure, reaction temperature, nature of the cocatalyst, catalyst dosage and substrate concentration on the molecular weight, yield and selectivity of the polymer were explored in detail. The results showed that the (salen-**4**)[Co(III)TFA]_2_/[PPN]TFA catalyst system demonstrated a remarkable TOF as high as 735 h^–1^. In addition, a hypothetical catalytic reaction mechanism was proposed based on density functional theory (DFT) calculations and the catalytic reaction results of the (salen-**4**)[Co(III)TFA]_2_.

## 1. Introduction

**Scheme 1:**
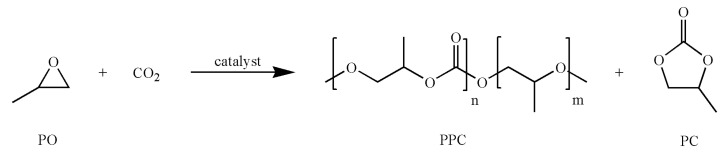
Reaction of propylene oxide (PO) and CO_2_ to yield poly(propylene carbonate) (PPC) and propylene carbonate (PC).

With the development of social industrialization, the concentration of CO_2_ in the atmosphere keeps rising, resulting in the greenhouse effect and a series of associated environmental disasters [1]. Nevertheless, CO_2_ is an inexpensive, a nontoxic, rich and special renewable C1 building block [2,3,4]. So far, wide attention has been paid to the chemical conversion of CO_2_ into high value-added energy, materials and chemical products. The beneficial use of CO_2_ including the alternating copolymerization of CO_2_ and epoxides to make degradable polycarbonates were popular (Scheme 1), which is considered one of the most potential green polymerization processes [5,6,7,8,9,10,11]. In addition, polycarbonates have become important topics because of their environment-friendly properties, such as atom-economy, energy-saving and degradation, as well as their several biomedical and pharmaceutical applications [12].

The problem is that CO_2_ is a highly stable and low-reactivity molecule, making it difficult to use CO_2_ as a reagent in a chemical reaction. Inspired by Inoue’s pioneering work, researchers have developed many catalysts [13,14,15,16,17,18,19,20,21,22,23]. Nevertheless, it is still a big challenge for scientists to develop low-cost, easily prepared and highly efficient catalysts for fixation and conversion of CO_2_ under mild conditions. Among them, salen metal complexes were widely used due to their easy modification, mild polymerization conditions, high activity and selectivity of poly(propylene carbonate) (PPC), excellent regioselectivity and stereoselectivity, as well as the high molecular weight with narrow molecular weight distribution PDI [24,25,26,27,28,29,30,31]. To improve the performance of salen-Co catalysts, structures with piperidinium end-capping arms, quaternary ammonium salts or other functional groups were explored [32,33,34,35,36].

Recently, dinuclear catalysts have aroused much attention. It is found that the two metal centers could work cooperatively in propylene oxide (PO) and CO_2_ copolymerization [37,38]. Nakano et al. synthesized a series of dinuclear salen-Co catalysts [9] and proposed a bimetallic mechanism for the dinuclear catalyst in the absence of cocatalyst. Klaus and coworkers developed a dinuclear salen-Cr complex. They found that with a suitable distance, the two metal centers showed a perfect synergy in high dilution, but a low selectivity of carbonate linkages [39]. Furthermore, in Li’s research, the optimal dinuclear catalyst showed a TOF of 254 h^–1^ at a [PO]/[Co] ratio of 2000, and the TOF increased to 406 h^–1^ at a [PO]/[Co] ratio of 20,000. In contrast, the TOF of the mononuclear catalyst system dropped from 674 h^–1^ to 3 h^–1^ upon dilution from 2500 to 20,000 [40]. Although these catalysts were impressive, they were less active and no high-molecular-weight PPC was obtained. 

**Figure 1:**
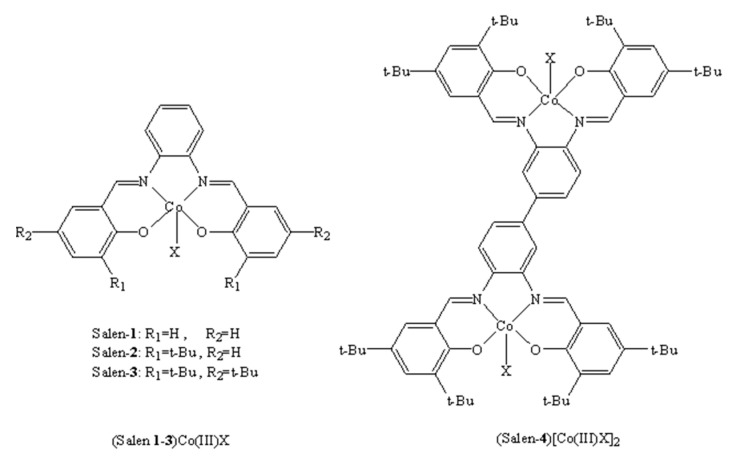
(Salen)Co(III)X catalyst for PO/CO_2_ copolymerization (X = Cl, DNP, TFA).

Previous studies have shown that reaction conditions play very important roles in binary systems [41]. Therefore, it is of great significance to explore the effect of reaction conditions on dinuclear catalytic activity. Inspired by Li’s efficient salph-co(III) catalysts [40], we firstly synthesized a new dinuclear complex (salen-**4**)[Co(III)TFA]_2_ (Figure 1). For comparison, we also synthesized a series of mononuclear complexes (salen **1**-**3**)Co(III)X (X = Cl, DNP = 2,4-dinitrophenol, TFA) and two dinuclear complexes (salen-**4**)[Co(III)Cl]_2_, (salen-**4**)[Co(III)DNP]_2_ (Figure 1). And the influences of reaction conditions (time, pressure, temperature, cocatalyst, catalyst dosage and substrate concentration) were explored in detail. When (salen-**4**)[Co(III)TFA]_2_ was used as a catalyst for PO and CO_2_ copolymerization under mild conditions, it displayed excellent catalytic performance with high activity and selectivity. Furthermore, the relationship between the molecular structures and catalysis performance was analyzed based on experiments and DFT calculations, and a reaction mechanism was proposed.

## 2. Results and Discussion

### 2.1. Synthesis and Characterization of (Salen-**4**)[Co(III)TFA]_2_

**Scheme 2:**
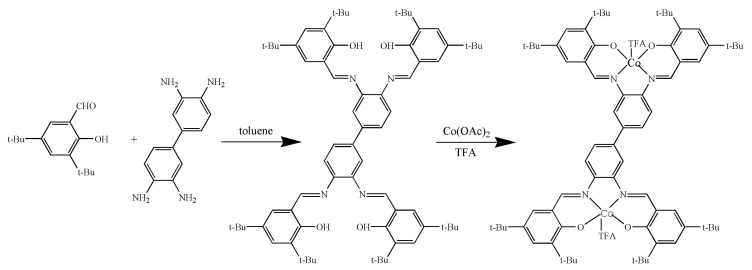
Synthesis of (salen-**4**)[Co(III)TFA]_2_.

Based on the ligand H_4_Salen-8*^t^*Bu (salen-**4**), a new dinuclear cobalt complex (salen-**4**)[Co(III)TFA]_2_ has been firstly synthesized and characterized. The synthesis of (salen-**4**)[Co(III)TFA]_2_ was achieved as shown in Scheme 2. Reaction of 3, 5-di-*tert*-butylsalicylaldehyde with 3,3′-diaminobiphenylamine gave ligand salen-**4**. Treatment of ligand salen-**4** with cobalt acetate gave bimetallic complexes (salen-**4**)[Co(II)]_2_, which when treated with trifluoroacetate under oxygen atmosphere gave the desired complex (salen-**4**)[Co(III)TFA]_2_.

**Figure 2:**
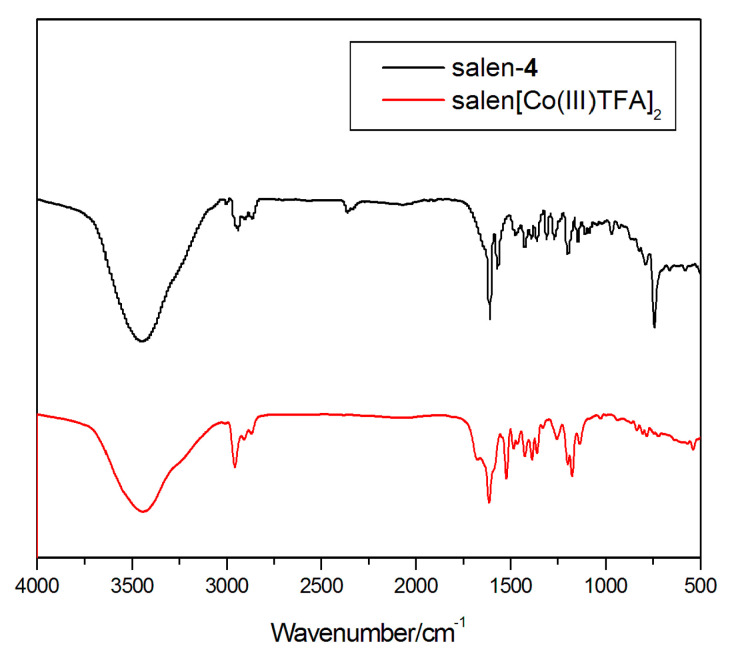
IR of ligand salen-**4** and complex (salen-**4**)[Co(III)TFA]_2_.

Complex (salen-**4**)[Co(III)TFA]_2_ was characterized by FT-IR (Figure 2). As is readily observed, compared with the ligand salen-**4**, 2957 cm^−1^ and 2870 cm^−1^ can be considered as the stretching vibration of C-H in the methyl group on the *tert*-butyl; 1682 cm^−1^ can be considered as the characteristic absorption peak of axial ligand CF_3_COO^−^. Due to the coordination of the ligand with Co(III), the characteristic absorption peak of the 1634 cm^−1^ *v(*C=N) in salen-**4** shifted to the lower wavenumber by 21 cm^−1^, and peaked at 1613 cm^−1^. Here, 1522 cm^−1^ and 1484 cm^−1^ can be considered as the skeleton vibration of the benzene, and 1387 cm^−1^ can be considered as the inplane bending vibration of the C-H on -CH_3_. Due to the drop of the hydrogen atom on Ph-OH during the coordination process, salen-**4** *v(*C-O) stretching vibration of Ph-OH at 1272 cm^−1^ is shifted by 16 cm^−1^ to the lower wavenumber, and the peak appeared at 1253 cm^−1^. Here, 1175 cm^−1^ can be considered as the C-H inplane bending vibration of the benzene and 783 cm^−1^ can be considered as the out-of-plane bending vibration of C-H on the benzene. Therefore, Salen-**4**[Co(III)TFA]_2_ was successfully synthesized.

### 2.2. (Salen)Co(III)X Catalyzed PO/CO_2_ Copolymerization

The copolymerization of PO/CO_2_ was conducted in the presence of various catalysts, and the results are listed in Table 1. We designed a series of experiments to explore the potential catalytic performance of (salen)Co(III)X. As shown in Table 1, the catalyst and the cocatalyst had a significant effect on the copolymerization of PO/CO_2_. Dinuclear complex (salen-**4**)[Co(III)DNP]_2_ showed higher activity and selectivity than (salen-**3**)Co(III)DNP (which is one half of the dinuclear (salen-**4**)[Co(III)DNP]_2_ complexes structurally) (Entries 4 and 7, Table 1). The conversion of PO was 99%, the selectivity of PPC was 91% and TOF was 225 h^–1^. Importantly, with [PPN]TFA as the cocatalyst, the activity of dinuclear complex (salen-**4**)[Co(III)TFA]_2_ was much higher than that of mononuclear complex (salen-**3**)Co(III)TFA (Entries 5 and 8, Table 1) with TOF reaching 230 h^–1^, which was two times of that for (salen-**3**)Co(III)TFA.

Interestingly, we found that as the number of *tert*-butyl groups with large steric hindrance on ligands of the complexes (Entries 1–3, Table 1) increased, the conversion of PO and selectivity of PPC both increased. With the rising of electron absorption capacity of axial groups of the complexes (Entries 3–5, Table 1), the conversion of PO increased continuously, so did the selectivity of PPC. Meanwhile, the dinuclear complexes (Entries 6–8, Table 1) showed the same results. By comparing mononuclear and dinuclear metal complexes of the same salen ligands and the same axial anions (Entries 3 and 6, 4 and 7, 5 and 8, Table 1), it was found that the conversion of PO and selectivity of PPC both increased slightly. The TOF also increased by two times, indicating that the dinuclear catalytic system was much more efficient than the mononuclear catalytic system for CO_2_ and PO copolymerization.

Furthermore, the optimal catalyst (salen-**4**)[Co(III)TFA]_2_ was selected. Next, the influences of reaction time, CO_2_ pressure, reaction temperature, cocatalyst, catalyst dosage and substrate concentration on PO/CO_2_ copolymerization were explored to improve the yield and selectivity of PPC.

#### 2.2.1. Effect of Reaction Temperature

The conversion of PO increased along with the rising temperature and remained stable until it reached 25 °C. By contrast, the selectivity of PPC increased first and then decreased (Table 2). That is because the binding of PO and metal center was blocked when the temperature was too low, which lowered the activation rate of PO, further resulting in the lower conversion of PO, the selectivity of PPC and polymerization rate. By contrast, with the rising temperature, the selectivity of PPC increased first and then decreased. That is because when the temperature is too high, the product tends to generate propylene carbonate (PC) with higher thermodynamic stability. It is known that the thermodynamic stability of PPC is lower than PC, which reduces the selectivity of PPC. Therefore, the optimal polymerization temperature of (salen-**4**)[Co(III)TFA]_2_/[PPN]TFA is 25 °C.

#### 2.2.2. Effect of CO_2_ Pressure

The conversion of PO and selectivity of PPC increased (Table 3) as the CO_2_ pressure rose. When the pressure of CO_2_ was too low, the combination of PO and metal center was blocked, thereby lowering the activation rate of PO, the conversion of PO, the selectivity of PPC and the polymerization rate. The yield of PPC increased along with the CO_2_ pressure. When the CO_2_ pressure exceeded 3.0 MPa, the yield of PPC did not increase significantly. Given that there is a potential safety hazard in laboratory operations when the CO_2_ pressure is too high, thus, the optimal polymerization pressure of (salen-**4**)[Co(III)TFA]_2_/[PPN]TFA is 3.0 MPa.

#### 2.2.3. Effect of Reaction Time 

With the increase of reaction time, the conversion of PO increased gradually (Table 4). When the reaction time exceeded 4 h, the conversion of PO did not change much. The possible reason is that when the reaction time is too long, the polymer will adhere to the surface of the catalyst and reduces the contact area between the active center and PO, resulting in a slower catalytic rate. This indicates that the catalytic system basically reached equilibrium after 4 h. Therefore, the optimal reaction time of (salen-**4**)[Co(III)TFA]_2_/[PPN]TFA is 4 h.

#### 2.2.4. Effect of Cocatalyst 

It was found that cocatalysts played very important roles in binary systems. During the CO_2_ copolymerization with PO, the CO_2_ pressure was 3.0 MPa, the reaction temperature was 25 °C, the reaction time lasted 4 h and n(PO)/n(Cat.)/n(Co-cat.) = 3000:1:1. The system with [PPN]TFA as the cocatalyst exhibited much higher catalytic activity than those with TBAB or [PPN]Cl as the cocatalyst (Table 5). Therefore, [PPN]TFA was selected as the best cocatalyst to form a two-component catalytic system.

#### 2.2.5. Effect of Substrate Concentration 

When n(Cat.)/n(Co-cat.) = 1:1, as the molar amount of reaction substrate increased, the conversion of PO increased and then decreased. When n(PO)/n(Cat.)/n(Co-cat.) = 3000:1:1 (Entry 2, Table 6), the conversion of PO and selectivity of PPC reached the maximum. However, when n(PO)/n(Cat.)/n(Co-cat.) = 3000:0.5:1 (Entry 3, Table 6), the conversion of PO and selectivity of PPC further increased and the TOF reached 735 h^–1^. It is probably because the (salen-**4**)[Co(III)TFA]_2_ as a dinuclear catalyst, has two metal active centers. When n(Cat.)/n(Co-cat.) = 1:2, the TFA anion in [PPN]TFA formed a 1:1 molar ratio to the active center, which accelerated the activation of PO as well as the chain growth of polymerization reaction, thereby leading to a further increase in the conversion of PO and selectivity of PPC. Therefore, the optimal substrate concentration for (salen-**4**)[Co(III)TFA]_2_/[PPN]TFA is n(PO)/n(Cat.)/n(Co-cat.) = 3000:0.5:1. In addition, when n(PO)/n(Cat.)/n(Co-cat.) = 10,000:1:1, the (salen-**4**)[Co(III)TFA]_2_/[PPN]TFA catalytic system still performed well, whereas, the mononuclear complex (salen-**3**)Co(III)TFA activity would be greatly reduced or even inactivated [42].

### 2.3. Theoretical Calculations of Quantum Chemistry

**Figure 3:**
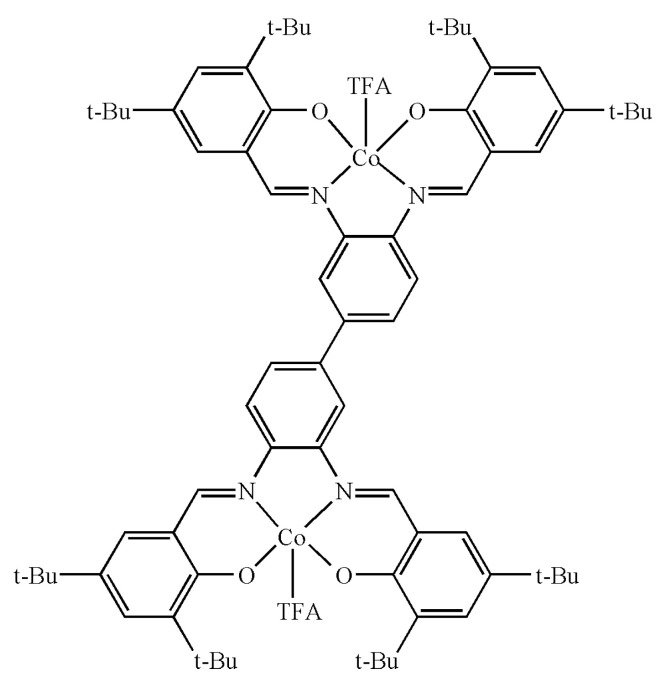
Calculation model of (salen-**4**)[Co(III)TFA]_2_.

In this part, (salen-**4**)[Co(III)TFA]_2_ was selected. In order to make the configuration simple and clear, the H atoms in the figure were omitted, but they were still included in the calculation process. Gaussian 16 [43] calculation program was used to calculate the (salen-**4**)[Co(III)TFA]_2_ model (Figure 3), and the DFT B3LYP method [44] was used to optimize the geometric configuration of the (salen-**4**)[Co(III)TFA]_2_ model at the level of pseudopotential group GENECP (the nonmetal atoms use the 6-31G** basis group [45] level, the Co atoms are heavy metal and the LanL2DZ basis group based on the effective core is adopted). 

**Figure 4:**
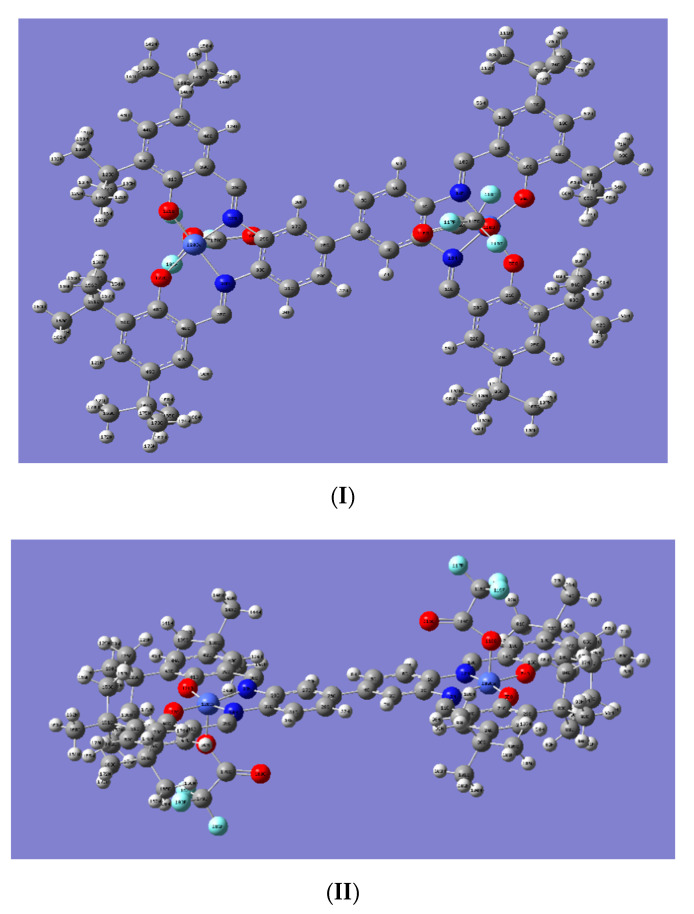
Structure of optimized (salen-**4**)[Co(III)TFA]_2_; (**I**) is the main view, (**II**) is the top view.

From the optimized geometry of (salen-**4**)[Co(III)TFA]_2_ (Figure 4), it can be seen that the two central metal Co atoms are in pentacoordinated structures [46] in which two N atoms and two O atoms of the ligand form the equatorial plane and the O atoms on the axial anion TFA occupy the axial position. Notably, the bond angles between the central metal Co atoms and the four coordination atoms of the ligand are about 90° or 180°, indicating that the Co atom is in a deformed planar quadrilateral field. The four bond angles formed by the metal Co atom and four coordination atoms on the ligand totals approximately about 360°, that is, the metal Co atom and the four coordination atoms are substantially in the same plane. In addition, the bond angles between the O atoms of TFA and metal Co atom, and the four coordination atoms on the ligand are about 90°. This indicates that the O atoms on the axial anion are basically perpendicular to the four coordination atoms on the ligand (Table S3). 

From the energy of the frontal molecular orbitals (Table 7), it can be seen that the occupied orbitals are all negative, suggesting that the electronic state of the complexes is stable and its photoelectron spectrum is meaningful [47]. It can also be seen that the order of the LUMO orbital energy of the complexes is: (salen-**4**)[Co(III)TFA]_2_ > (salen-**4**)[Co(III)DNP]_2_ > (salen-**4**)[Co(III)Cl]_2_, but the stability of the system after receiving electrons is in the opposite order. By comparing the magnitude of the energy difference ΔE (ΔE = E_LUMO_ − E_HOMO_) of the complexes, it is found that there is: (salen-**4**)[Co(III)Cl]_2_ > (salen-**4**)[Co(III)DNP]_2_ > (salen-**4**)[Co(III)TFA]_2_. The larger ΔE, the harder it is to excite the electrons of the molecule, the more stable the molecule and the weaker the activity. Therefore, the order of the ability of the complexes to accept the lone pair of electrons of O on PO is: (salen-**4**)[Co(III)TFA]_2_ > (salen-**4**)[Co(III)DNP]_2_ > (salen-**4**)[Co(III)Cl]_2_. In other words, (salen-**4**)[Co(III)TFA]_2_ has the strongest ability to activate PO, which affects the catalytic reaction activity and is consistent with the results of catalytic experiments.

### 2.4. Proposed Mechanism for the Catalyst

**Figure 5:**
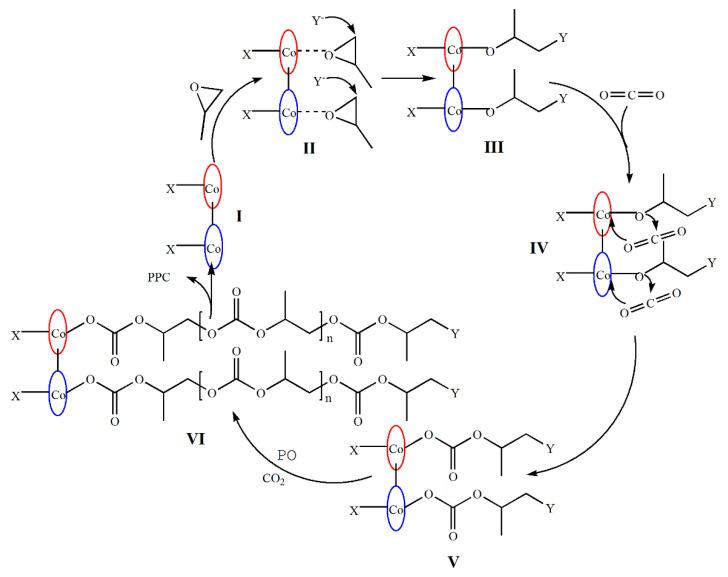
Proposed mechanism of CO_2_/PO copolymerization with the dinuclear catalyst.

Based on the above research, we proposed mechanisms for the dinuclear catalyst systems. In previous reports, a great deal of supported that CO_2_ “insertion” actually occurs via a dissociative mechanism where a free nucleophilic chain end (alkoxide) attacks the CO_2_ reversibly to give a carbonate end that is stabilized by the charge of the metal complex [16,37,41,48,49,50,51]. As shown in Figure 5, first, the dinuclear complex interacted with the cocatalyst [PPN]Y to form two active centers (**I**), and the two active centers simultaneously activated the attacking PO to form a pair of hexacoordinate structures (intermediate **II**). Subsequently, the nucleophile Y^−^ attacked the C atom with a smaller steric hindrance on PO. Then, CO_2_ was inserted into the metal alkoxide bond (intermediate **III**), forming the carbonate unit (intermediate **IV**). The growing copolymer chain could migrate to the other mental center and attack the activated PO (intermediate **V**), which was supposed to be the key process for the bimetallic synergism. In the chain growth stage: CO_2_ and PO were alternately copolymerized and M-O was broken to form poly(propylene carbonate) (**VI**) finally. The two active sites activated CO_2_ and PO at the same time and greatly improved the activation rate, which also explained the reason why the catalytic performance of the dinuclear system was significantly better than that of the mononuclear system.

## 3. Materials and Methods 

All reagents and solvents were analytical grade and were ready to use. The ligands H_2_Salen (salen-**1**), H_2_Salen-2*^t^*Bu (salen-**2**), H_2_Salen-4*^t^*Bu (salen-**3**), H_4_Salen-8*^t^*Bu (salen-**4**) were synthesized following the reported procedure [41,52,53]. Propylene oxide was refluxed over a mixture of KOH/CaH_2_, and fractionally distilled under an argon atmosphere prior to use. Carbon dioxide (99.99%) and oxygen (99.99%) were purchased from Shaanxi Heping Glass Co., Ltd. (Shaanxi, China). and used as received. All other reagents were purchased from commercial sources and used as received. FT-IR spectra (Madison, WI, USA) were obtained on a VERTEX-70 Fourier transform infrared spectrometer with a band ranging from 4000 to 400 cm^–1^. The elemental analysis of complexes was performed by Elementar Germany’s Vario MICRO elemental analyzer (Hanau, Germany). The UV spectrum data of complexes were obtained by a Japan Shimadzu UV-2600 ultraviolet spectrophotometer (Kyoto, Japan). NMR spectra were recorded on a JEOL ECS400M spectrometer (Tokyo, Japan) with reference to the solvent signals.

### 3.1. Catalyst Preparation

#### 3.1.1. Synthesis of Ligands 

H_2_Salen (salen-**1**). Under nitrogen atmosphere, o-phenylenediamine (7.03 g, 65 mmol) was added into a three-port flask containing 100 mL anhydrous ethanol. The flask was heated to reflux. After o-phenylenediamine was completely dissolved, salicylaldehyde (1.22 g, 10 mmol) was dropped into the flask. After 12 h, the mixture was cooled to room temperature and then transferred into a clean beaker for standing. A large number of orange crystals precipitated and were collected after being filtered, washed with anhydrous ethanol and dried in vacuo. Yield (based on *o*-phenylenediamine): 82%. EA (%) C_20_H_16_N_2_O_2_: calc. C 75.92, H 5.11, N 8.85, found C 75.98, H 5.04, N 8.81. UV-Vis (CH_3_Cl_3_) λ_max_/nm (ε/L^.^mol^–1.^cm^–1^): 240 (0.23 × 105), 267 (0.29 × 105), 334 (0.23 × 105). ^1^H NMR (400 MHz, CDCl_3_, TMS) δ (ppm): 6.90 (t, 2H, Ar-H), 7.04 (d, 2H, Ar-H), 7.21 (q, 2H, Ar-H), 7.24 (s, 2H, Ar-H), 7.32 (q, 2H, Ar-H), 7.35 (s, 2H, Ar-H), 8.61 (s, 2H, N=C-H), 13.04 (s, 2H, -OH). IR (KBr, *v*, cm^–1^): 3448(s), 2930(w), 2780(w), 1630(s), 1566(m), 1478(s), 1388(s), 1278(s), 1180(s), 762(s). 

H_2_Salen-2*^t^*Bu (salen-**2**). Salen-**2** was obtained with the similar procedure for salen-**1** except that salicylaldehyde was replaced by 3-*tert*-butylsalicylaldehyde (1.78 g, 10 mmol). Yield (based on o-phenylenediamine): 79%. EA (%) C_28_H_32_N_2_O_2_: calc. C 78.46, H 7.54, N 6.53, found C 78.51, H 7.52, N 6.48. UV-Vis (CH_3_Cl_3_) λ_max_/nm (ε/L^.^mol^–1.^cm^–1^): 241 (0.24 × 105), 275 (0.31 × 105), 366 (0.24 × 105). ^1^H NMR (400 MHz, CDCl_3_, TMS) δ (ppm): 1.43 (t, 18H, *t*-Bu), 6.85 (t, 2H, -OH), 7.23 (t, 2H, Ar-H), 7.25 (s, 2H, Ar-H), 7.31 (d, 2H, Ar-H), 7.34 (t, 2H, Ar-H), 7.39 (d, 2H, Ar-H), 8.66 (s, 2H, N=C-H). IR (KBr, *v*, cm^–1^): 3450(s), 3010(w), 2948(m), 2867(w), 1628(s), 1566(s), 1482(m), 1432(m), 1368(m), 1268(s), 1191(s), 753(s). 

H_2_Salen-4*^t^*Bu (salen-**3**). Salen-**3** was obtained with the similar procedure for salen-**1** except that salicylaldehyde was replaced by 3, 5-di-*tert*-butylsalicylaldehyde (2.34 g, 10 mmol). Yield (based on o-phenylenediamine): 75%. EA (%) C_36_H_48_N_2_O_2_: calc. C 79.94, H 8.96, N 5.18, found C 80.01, H 9.01, N 5.16. UV-Vis (CH_3_Cl_3_) λ_max_/nm (ε/L^.^mol^–1.^cm^–1^): 242 (0.23 × 105), 279 (0.22 × 105), 338 (0.16 × 105). ^1^H NMR (400 MHz, CDCl_3_, TMS) δ (ppm): 1.33 (t, 18H, *t*-Bu), 1.44 (t, 18H, *t*-Bu), 7.21 (d, 2H, -OH), 7.22 (t, 2H, Ar-H), 7.25 (d, 2H, Ar-H), 7.31 (t, 2H, Ar-H), 7.44 (d, 2H, Ar-H), 8.67 (s, 2H, N=C-H). IR (KBr, *v*, cm^–1^): 3452(s), 2961(m), 2866(w), 1634(s), 1566(w), 1477(w), 1438(w), 1382(s), 1272(m), 1172(s), 753(m). 

H_4_Salen-8*^t^*Bu (salen-**4**). Under nitrogen atmosphere, 3, 3′-diaminobenzidine (2.5 g, 11.67 mmol), 3, 5-di-*tert*-butylsalicylaldehyde (15 g, 64.01 mmol) and p-toluenesulfonic acid (0.1 g, 0.58 mmol) were added into a 1000 mL flask. Freshly distilled toluene was added to set up an azeotropic dewatering device. After 72 h, the solvent was removed in vacuo and solids were washed with ethanol. The mixture was recrystallized by dissolving it in methylene chloride and tetrahydrofuran, respectively. A high-purity ligand was obtained. Yield (based on 3, 3′-diaminobenzidine): 60%. EA (%) C_72_H_94_N_4_O_4_: calc. C 80.09, H 8.79, N 5.19, found C 80.14, H 8.86, N 5.12. UV-Vis (CH_3_Cl_3_) λ_max_/nm (ε/L^.^mol^–1.^cm^–1^): 230 (0.95 × 105), 281 (0.58 × 105), 365 (0.54 × 105). ^1^H NMR (400 MHz, CDCl_3_, TMS) δ (ppm): 1.32 (s, 36H, *t*-Bu), 7.15~7.62 (m, 50H, Ar-H), 8.44 (s, 4H, N=C-H), 11.51 (s, 4H, -OH). IR (KBr, *v*, cm^–1^): 3448(s), 2953(s), 2864(w), 1635(s), 1580(w), 1471(m), 1432(m), 1384(m), 1269(w), 1168(s), 768(w). 

#### 3.1.2. Synthesis of Complexes

(Salen-**1**)Co(III)Cl. The ligand salen-**1** (0.07 g, 0.22 mmol) and decrystallized water Co(OAc)_2_ (0.05 g, 0.27 mmol) were dissolved in 10 mL anhydrous methanol. The mixture was stirred at room temperature for 12 h, and then anhydrous LiCl (0.05 g, 1.10 mmol) was added and passed in oxygen to continue the reaction for 12 h. Solvent was removed under reduced pressure and the residue was dissolved in CH_2_Cl_2_. The organic layer was rinsed with saturated aqueous Na_2_SO_4_ (50 mL × 3) and NaCl (50 mL × 3), respectively. The organic layer was dried over Na_2_SO_4_, filtered and dried in vacuo. The crude product was recrystallized from CH_2_Cl_2_ and anhydrous CH_3_CH_2_OH to obtain a brown-red solid [54]. Yield: 82%. EA (%) C_20_H_14_CoN_2_O_2_Cl: calc. C 58.77, H 3.45, N 6.85, found C 58.83, H 3.48, N 6.81. UV-Vis (CH_3_Cl_3_) λ_max_/nm (ε/L^.^mol^–1.^cm^–1^): 254 (0.41 × 105), 369 (0.11 × 105), 387 (0.11 × 105), 473 (0.08 × 105). IR (KBr, *v*, cm^–1^): 3440(s), 2920(w), 2850(w), 1614(s), 1560(m), 1477(s), 1386(s), 1269(s), 1145(s), 762(s). 

(Salen-**2**)Co(III)Cl. (Salen-**2**)Co(III)Cl was obtained with the similar procedure for (salen-**1**)Co(III)Cl except that ligand salen-**1** was replaced by ligand salen-**2** (0.09 g, 0.22 mmol). Yield: 75%. EA (%) C_28_H_30_CoN_2_O_2_Cl: calc. C 64.56, H 5.80, N 5.38, found C 64.49, H 5.73, N 5.43. UV-Vis (CH_3_Cl_3_) λ_max_/nm (ε/L^.^mol^–1.^cm^–1^): 260 (0.40 × 105), 369 (0.14 × 105), 391 (0.16 × 105), 508 (0.07 × 105). IR (KBr, *v*, cm^–1^): 3440(s), 2949(m), 2863(w), 1613(s), 1527(s), 1489(m), 1387(s), 1261(w), 1145(s), 741(s).

(Salen-**3**)Co(III)Cl. (Salen-**3**)Co(III)Cl was obtained with the similar procedure for (salen-**1**)Co(III)Cl except that ligand salen-**1** was replaced by ligand salen-**3** (0.12 g, 0.22 mmol). Yield: 72%. EA (%) C_36_H_46_CoN_2_O_2_Cl: calc. C 68.29, H 7.32, N 4.42, found C 68.23, H 7.40, N 4.39. UV-Vis (CH_3_Cl_3_) λ_max_/nm (ε/L^.^mol^–1.^cm^–1^): 264 (0.27 × 105), 369 (0.11 × 105), 411 (0.08 × 105), 513 (0.05 × 105). IR (KBr, *v*, cm^–1^): 3450(s), 2955(s), 2860(w), 1610(s), 1527(s), 1489(w), 1387(s), 1261(w), 1145(w), 743(s).

(Salen-**3**)Co(III)DNP. The ligand salen-**3** (1.00 g, 1.85 mmol) and Co(OAc)_2_^.^4H_2_O (0.33 g, 1.3 mmol) were dissolved in 100 mL absolute ethanol. The mixture was heated to 80 °C for 30 min and then cooled to room temperature. The solvent was removed in vacuo and washed with cold methanol (15 mL × 3). The solid was dissolved in 10 mL CH_2_Cl_2_ and recrystallized by 200 mL *n*-hexane. After 24 h, a dark red crystal (salen-**4**)Co(II) was obtained. The (salen-**4**)Co(II) (0.07 g, 0.12 mmol) was dissolved in 50 mL CH_2_Cl_2_ and 2, 4-dinitrophenol sodium (0.02 g, 0.12 mmol) was added. The resulting solution was stirred for 2 h at room temperature in an oxygen atmosphere. Solvent was removed in vacuo to obtain the black crude product. The crude product was recrystallized from CH_2_Cl_2_ and *n*-hexane, respectively, and then dried in vacuo at 60 °C for 24 h [40]. Yield: 71%. EA (%) C_42_H_49_CoN_4_O_7_: calc. C 64.61, H 6.33, N 7.18, found C 64.53, H 6.29, N 7.14. UV-Vis (CH_3_Cl_3_) λ_max_/nm (ε/L^.^mol^–1.^cm^–1^): 263 (0.48 × 105), 321 (0.33 × 105), 430 (0.30 × 105), 533 (0.17 × 105). IR (KBr, *v*, cm^–1^): 3450(s), 2955(m), 2866(w), 1615(s), 1572(w), 1521(s), 1457(m), 1387(s), 1257(s), 1170(s), 753(w).

(Salen-**3**)Co(III)TFA. (Salen-**3**)Co(III)Cl (0.08 g, 0.12 mmol) and CF_3_COOAg (0.03 g, 0.12 mmol) were dissolved in 15 mL CH_2_Cl_2_. Under oxygen atmosphere, the mixture was stirred for 24 h in the dark. Solvent was removed by vacuum filtration. A red-black solid was obtained and recrystallized from CH_2_Cl_2_ and *n*-hexane, respectively [55]. Yield: 73%. EA (%) C_38_H_46_CoN_2_O_4_F_3_: calc. C 64.22, H 6.52, N 3.94, found C 64.29, H 6.61, N 4.01. UV-Vis (CH_3_Cl_3_) λ_max_/nm (ε/L^.^mol^–1.^cm^–1^): 263 (0.40 × 105), 308 (0.14 × 105), 401 (0.16 × 105), 506 (0.07 × 105). IR (KBr, *v*, cm^–1^): 3450(s), 2955(s), 2866(m), 1687(m), 1628(s), 1526(s), 1459(s), 1387(s), 1252(s), 1182(s), 753(m).

(Salen-**4**)[Co(III)Cl]_2_. The ligand salen-**4** (0.24 g, 0.22 mmol) was dissolved in 100 mL anhydrous methanol. The mixture was heated to reflux at 65 °C. After the ligand was completely dissolved, decrystallized water Co(OAc)_2_ (0.09 g, 0.50 mmol) was dissolved in. Under oxygen atmosphere, anhydrous LiCl (0.02 g, 0.50 mmol) was added. After 12 h, the solvent was removed in vacuo. The residue was dissolved in CH_2_Cl_2_ and recrystallized by anhydrous CH_3_CH_2_OH. The resulting solution was filtered and washed to obtain a brown-red solid [40]. Yield: 82%. EA (%) C_72_H_90_Co_2_N_4_O_4_Cl_2_: calc. C 68.40, H 7.18, N 4.43, found C 68.46, H 7.23, N 4.49. UV-Vis (CH_3_Cl_3_) λ_max_/nm (ε/L^.^mol^–1.^cm^–1^): 232 (1.02 × 105), 268 (0.72 × 105), 421 (0.17 × 105), 513 (0.12 × 105). IR (KBr, *v*, cm^–1^): 3440(s), 2955(s), 2859(w), 1610(s), 1515(s), 1489(m), 1381(s), 1259(s), 1171(s), 761(m).

(Salen-**4**)[Co(III)DNP]_2_. Under nitrogen atmosphere, the ligand salen-**4** (2.16 g, 2.0 mmol) was dissolved in 20 mL CH_2_Cl_2_, and Co(OAc)_2_ (0.72 g, 4.04 mmol) was dissolved in 80 mL anhydrous methanol. The mixture was stirred for 3 h at room temperature to obtain the (salen-**4**)Co(II) complex. Under oxygen atmosphere, (salen-**4**)Co(II) (1.20 g, 1.0 mmol) was dissolved in 50 mL CH_2_Cl_2_, and 2, 4-dinitrophenol sodium (0.41 g, 2.0 mmol) was added. The mixture was stirred for 3 h. Solvent was removed in vacuo to obtain the crude product. The crude product was recrystallized from CH_2_Cl_2_ and *n*-hexane, respectively to obtain a dark green solid [40]. Yield: 76%. EA (%) C_42_H_49_Co_2_N_4_O_7_: calc. C 68.69, H 6.20, N 7.18, found C 68.60, H 6.13, N 7.09. UV-Vis (CH_3_Cl_3_) λ_max_/nm (ε/L^.^mol^–1.^cm^–1^): 235 (1.07 × 105), 270 (1.00 × 105), 418 (0.47 × 105), 533 (0.26 × 105). IR (KBr, *v*, cm^–1^): 3443(s), 2955(s), 2868(w), 1614(s), 1573(m), 1522(s), 1483(m), 1386(s), 1253(s), 1197(s), 783(m).

(Salen-**4**)[Co(III)TFA]_2_. (Salen-**3**)Co(III)TFA (0.09 g, 0.12 mmol) and CF_3_COOAg (0.06 g, 0.25 mmol) were dissolved in 15 mL CH_2_Cl_2_. Under oxygen atmosphere, the mixture was stirred for 24 h in dark and filtered to remove the insoluble substances. Solvent was removed in vacuo to obtain a red-black solid. The solid was recrystallized from CH_2_Cl_2_ and *n*-hexane, respectively. Yield: 68%. EA (%) C_76_H_90_Co_2_N_4_O_8_F_6_: calc. C 64.31, H 6.39, N 3.95, found C 64.41, H 6.32, N 3.88. UV-Vis (CH_3_Cl_3_) λ_max_/nm (ε/L^.^mol^–1.^cm^–1^): 231 (1.01 × 105), 269 (0.68 × 105), 425 (0.22 × 105), 519 (0.17 × 105). ^1^H NMR (400 MHz, CDCl_3_, TMS) δ (ppm): 1.33 (s, 36H, *t*-Bu), 1.43 (s, 36H, *t*-Bu), 6.99 (s, 6H, Ar-H), 7.26 (s, 4H, Ar-H), 7.34 (s, 4H, Ar-H), 7.79 ((s, 4H, C = N-H). IR (KBr, *v*, cm^–1^): 3453(s), 2957(s), 2870(w), 1682(w), 1613(s), 1522(s), 1484(m), 1387(s), 1253(s), 1175(s), 783(m).

### 3.2. Catalytic Procedure

The catalyst, cocatalyst and epoxide were sequentially added into a 100 mL high-pressure reactor, and then the reactor was pressurized to a required pressure with CO_2_. The mixture was heated and stirred for a while. After completion of the reaction, unreacted CO_2_ was evacuated to remove the pressure. A small aliquot of the resultant polymerization mixture was removed from the reactor for ^1^H NMR and GPC analysis. The remaining polymerization mixture was then dissolved in tetrahydrofuran, quenched with 5% HCl solution in absolute ethanol, and precipitated from absolute ethanol (30 mL). The polymer was collected and dried in vacuo to constant weight, and the polymer yield was determined.

## 4. Conclusions

In summary, we have compared the mononuclear and dinuclear catalysts systems in PO and CO_2_ copolymerization. The dinuclear catalyst system has higher activity, higher selectivity and PPC with higher molecular weight. A mechanism based on the conjugated dinuclear structure was proposed. At the same time, the effects of reaction temperature, CO_2_ pressure, reaction time, catalyst’s type and substrate concentration on the dinuclear catalytic system were studied. The DFT method was used to optimize the geometries of the complexes. Moreover, the stable structure of the complexes was further recognized in theory and its effect on the catalytic reaction was analyzed. Furthermore, an ideal catalyst model for PO/CO_2_ copolymerization was constructed. The greater the number of *tert*-butyl groups on the ligand and the greater the anion electronegativity of the axial ligand, the higher the catalytic activity of the complex. The activity of the catalysts is expected to be further improved by changing the steric hindrances and electronic structures of the ligands, which will be further studied in the future.

## Figures and Tables

**Table 1:** (Salen)Co(III)X catalyzed PO/CO_2_ copolymerization ^a^.

Entry	Cat.	Co-cat.	T (h)	Con. ^b^ (%)	TOF ^c^ (h^–1^)	PPC ^d^ (%)
1	(salen-**1**)Co(III)Cl	[PPN]TFA	12	93	93	80
2	(salen-**2**)Co(III)Cl	[PPN]TFA	12	96	98	82
3	(salen-**3**)Co(III)Cl	[PPN]TFA	12	97	103	85
4	(salen-**3**)Co(III)DNP	[PPN]TFA	12	98	108	88
5	(salen-**3**)Co(III)TFA	[PPN]TFA	12	98	110	90
6	(salen-**4**)[Co(III)Cl]_2_	[PPN]TFA	6	99	223	90
7	(salen-**4**)[Co(III)DNP]_2_	[PPN]TFA	6	99	225	91
8	(salen-**4**)[Co(III)TFA]_2_	[PPN]TFA	6	99	230	93

^a^ Reaction conditions: 50 °C, 3.0 MPa, Entries 1–5: n(PO)/n(Cat.)/n(Co-cat.) = 1500:1:1, Entries 6–8: n(PO)/n(Cat.)/n(Co-cat.) = 3000:1:1. ^b^ Determined by ^1^H NMR analysis. ^c^ TOF = mol of product/mol of cobalt center per hour. ^d^ Selectivity for polycarbonates over cyclic carbonate, determined by ^1^H NMR spectroscopy.

**Table 2:** (Salen-**4**)[Co(III)TFA]_2_/[PPN]TFA catalyzed PO/CO_2_ copolymerization at different temperatures ^a^.

Entry	T (°C)	Con. ^b^ (%)	TOF ^c^ (h^–1^)	PPC ^d^ (%)
1	20	95	214	90
2	25	99	233	94
3	35	98	225	92
4	45	99	223	90
5	50	99	230	93

^a^ Reaction conditions: n(PO)/n(Cat.)/n(Co-cat.) = 3000:1:1, 6 h, 3.0 MPa. ^b^ Determined by ^1^H NMR analysis. ^c^ TOF = mol of product/mol of cobalt center per hour. ^d^ Selectivity for polycarbonates over cyclic carbonate, determined by ^1^H NMR spectroscopy.

**Table 3:** (Salen-**4**)[Co(III)TFA]_2_/[PPN]TFA catalyzed PO/CO_2_ copolymerization under different pressure ^a^.

Entry	P (MPa)	Con. ^b^ (%)	TOF ^c^ (h^–1^)	PPC ^d^ (%)
1	2.0	94	209	89
2	2.5	96	218	91
3	3.0	99	233	94
4	3.5	99	233	94

^a^ Reaction conditions: n(PO)/n(Cat.)/n(Co-cat.) = 3000:1:1, 6 h, 25 °C. ^b^ Determined by ^1^H NMR analysis. ^c^ TOF = mol of product/mol of cobalt center per hour. ^d^ Selectivity for polycarbonates over cyclic carbonate, determined by ^1^H NMR spectroscopy.

**Table 4:** (Salen-**4**)[Co(III)TFA]_2_/[PPN]TFA catalyzed PO/CO_2_ copolymerization at different reaction times ^a^.

Entry	T (h)	Con. ^b^ (%)	TOF ^c^ (h^–1^)	PPC ^d^ (%)
1	1	26	168	43
2	2	50	300	80
3	4	98	349	95
4	6	99	233	94

^a^ Reaction conditions: n(PO)/n(Cat.)/n(Co-cat.) = 3000:1:1, 3.0 MPa, 25 °C. ^b^ Determined by ^1^H NMR analysis. ^c^ TOF = mol of product/mol of cobalt center per hour. ^d^ Selectivity for polycarbonates over cyclic carbonate, determined by ^1^H NMR spectroscopy.

**Table 5:** (Salen-**4**)[Co(III)TFA]_2_ catalyzed PO/CO_2_ copolymerization under different cocatalyst ^a^.

Entry	Co-cat.	Con. ^b^ (%)	TOF ^c^ (h^–1^)	PPC ^d^ (%)
1	[PPN]Cl	82	194	63
2	TBAB	90	253	75
3	[PPN]TFA	98	349	95

^a^ Reaction conditions: n(PO)/n(Cat.)/n(Co-cat.) = 3000:1:1, 3.0 MPa, 25 °C, 4 h. ^b^ Determined by ^1^H NMR analysis. ^c^ TOF = mol of product/mol of cobalt center per hour. ^d^ Selectivity for polycarbonates over cyclic carbonate, determined by ^1^H NMR spectroscopy.

**Table 6:** (Salen-**4**)[Co(III)TFA]_2_/[PPN]TFA catalyzed PO/CO_2_ copolymerization at different substrate concentrations ^a^.

Entry	n(PO)/n(Cat.)/n(Co-cat.)	Con. ^b^ (%)	TOF ^c^ (h^–1^)	PPC ^d^ (%)
1	2000:1:1	94	216	92
2	3000:1:1	98	349	95
3	5000:1:1	45	127	45
4	10,000:1:1	30	113	30
5	3000:0.5:1	99	735	99

^a^ Reaction conditions: 3.0 MPa, 25 °C, 4 h. ^b^ Determined by ^1^H NMR analysis. ^c^ TOF = mol of product/mol of cobalt center per hour. ^d^ Selectivity for polycarbonates over cyclic carbonate, determined by ^1^H NMR spectroscopy.

**Table 7:** Frontline molecular orbital energy.

	E_HOMO_	E_LUMO_	ΔE
(salen-**4**)[Co(III)Cl]_2_	−0.21691	−0.11451	0.10240
(salen-**4**)[Co(III)DNP]_2_	−0.21061	−0.11252	0.09809
(salen-**4**)[Co(III)TFA]_2_	−0.19310	−0.10595	0.08715

## Supplementary Materials

The following are available online. Figures S1–S4: Infrared spectrum characterization of ligands and complexes, Figures S5–S8: Ultraviolet spectrum characterization of ligands and complexes, Figures S9–S12: ^1^H NMR spectrum characterization of ligands and complexes, Figures S13–S17: Characterization of the crude product PPC, Table S1: GPC of the crude product PPC, Table S2: Bond lengths [Å] and angle [°] after optimizing for (salen-**4**)[Co(III)TFA]_2_, Table S3: NBO charges distribution of some atoms in complex (salen-**4**)[Co(III)X]_2_.
