# Supplementary material for: A New Dinuclear Cobalt Complex for Copolymerization of CO2 and Propylene Oxide: High Activity and Selectivity
Source: Molecules. 2020 Sep 8;25(18):4095. doi: 10.3390/molecules25184095 (PMC7571228; doi:10.3390/molecules25184095)
Supplement: Supplementary file 1 [file molecules-25-04095-s001.pdf]

Supporting Information

A New Dinuclear Cobalt Complex for Copolymerization of CO_2 and Propylene Oxide: High Activity and Selectivity

Wen-zhen Wang,[*, a] Kai-yue Zhang,[a] Xin-gang Jia,[a] Li Wang,[a] Lei-lei Li,[a] Wei Fan[a] and Li Xia[a]

[a] *College of Chemistry and Chemical Engineering, Xi'an Shiyou University, Xi'an 710065, P. R. China*

[*] *E-mail: wzwang@xsyu.edu.cn.*

Contents

1. Infrared spectrum characterization. S2

2. Ultraviolet spectrum characterization. S3

3. ^{1}H NMR spectrum characterization. S5

4. Characterization of the crude product PPC. S7

5. The detailed quantum chemical theory calculation data. S10

1. Infrared spectrum characterization.

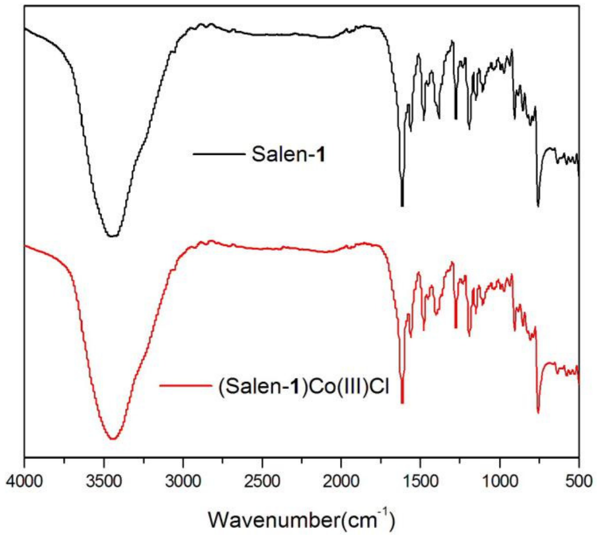

Figure S1. IR of ligand salen-**1** and complex (salen-**1**)Co(III)Cl.

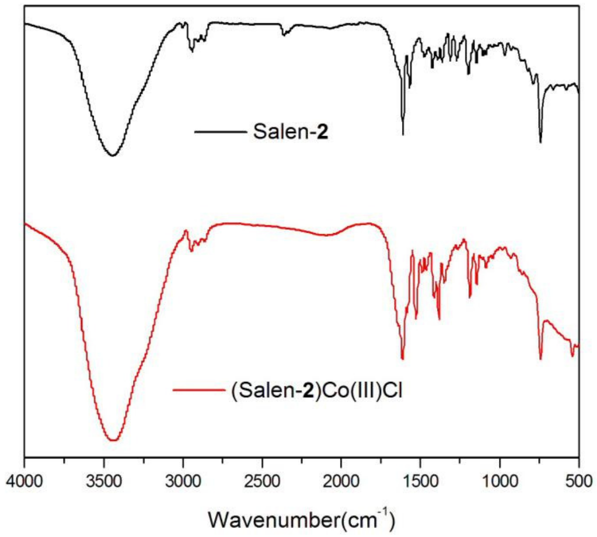

Figure S2. IR of ligand salen-**2** and (salen-**2**)Co(III)Cl.

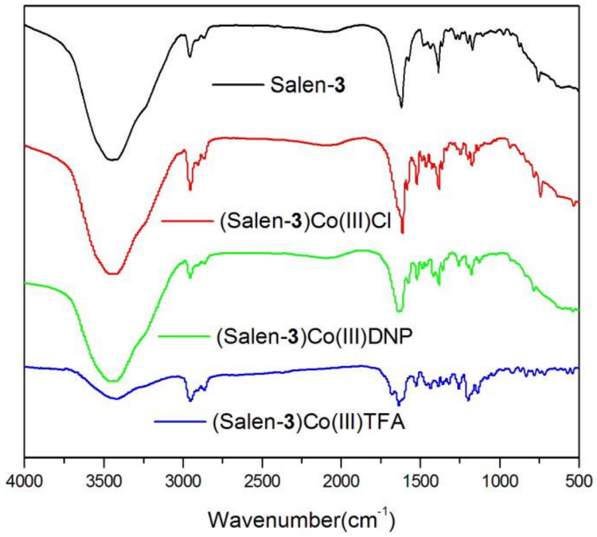

Figure S3. IR of ligand salen-**3** and complex (salen-**3**)Co(III)X (X =Cl, DNP, TFA).

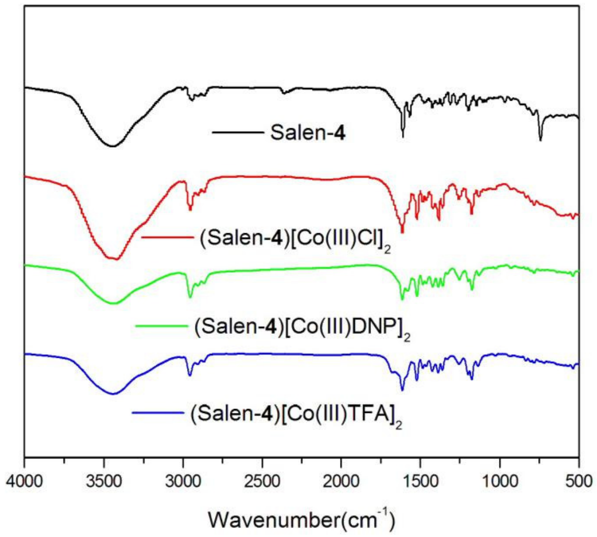

Figure S4. IR of ligand salen-**4** and complex (salen-**4**)$[Co(III)X]_2$ (X =Cl, DNP, TFA).

2. Ultraviolet spectrum characterization.

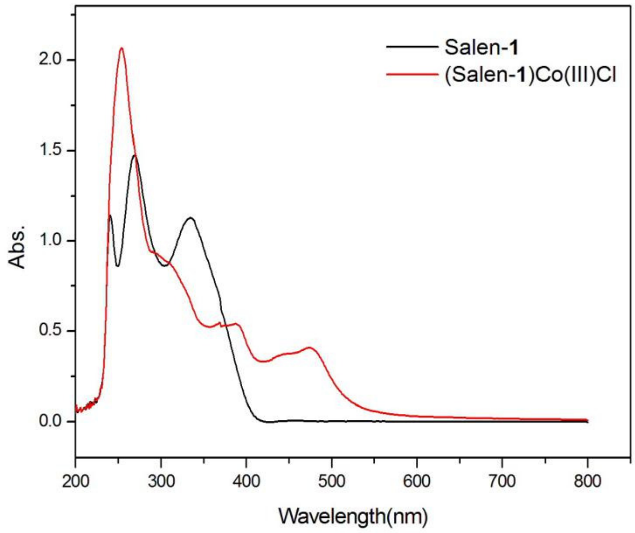

Figure S5. UV of ligand salen-**1** and complex (salen-**1**)Co(III)Cl.

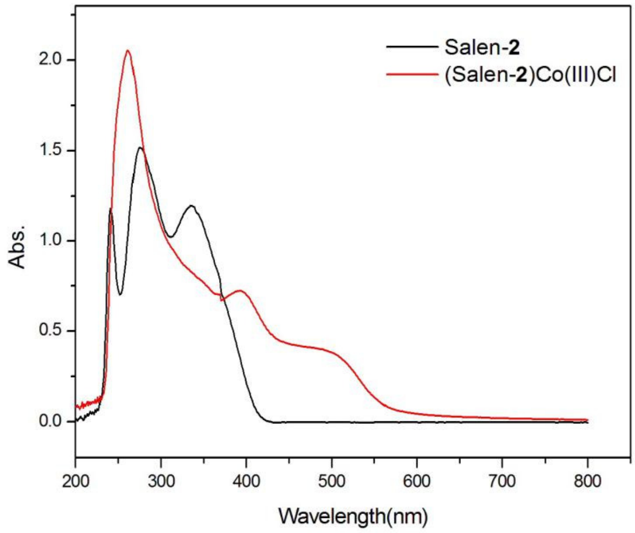

Figure S6. UV of ligand salen-**2** and complex (salen-**2**)Co(III)Cl.

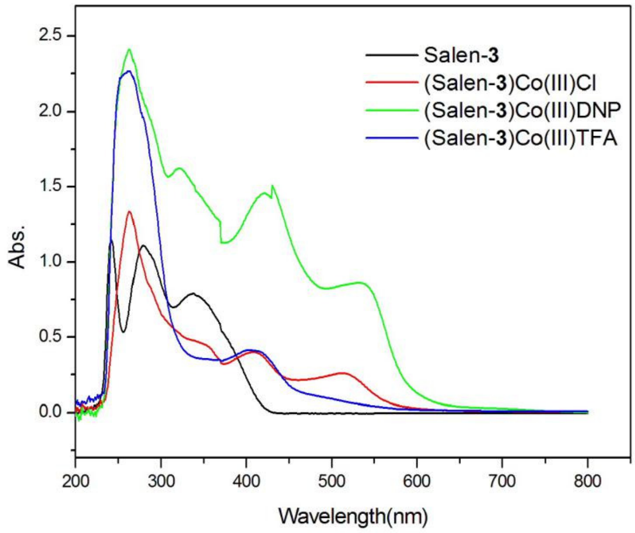

Figure S7. UV of ligand salen-**3** and complex (salen-**3**)Co(III)X (X=Cl, DNP, TFA).

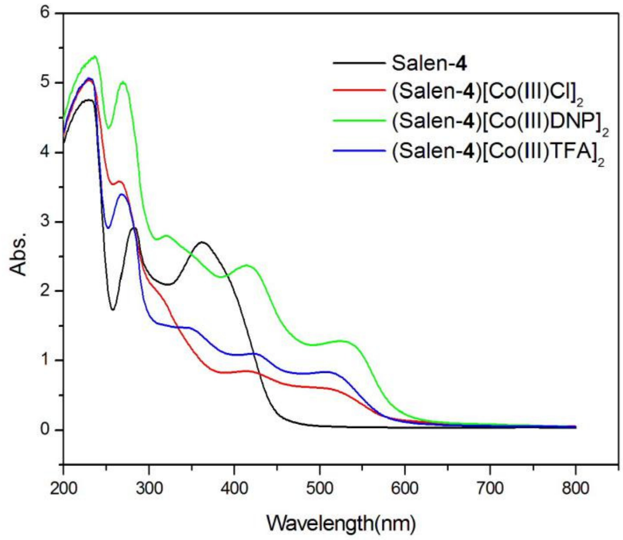

Figure S8. UV of ligand salen-**4** and complex (salen-**4**)[Co(III)X]$_2$ (X=Cl, DNP, TFA).

3. ^{1}H NMR spectrum characterization.

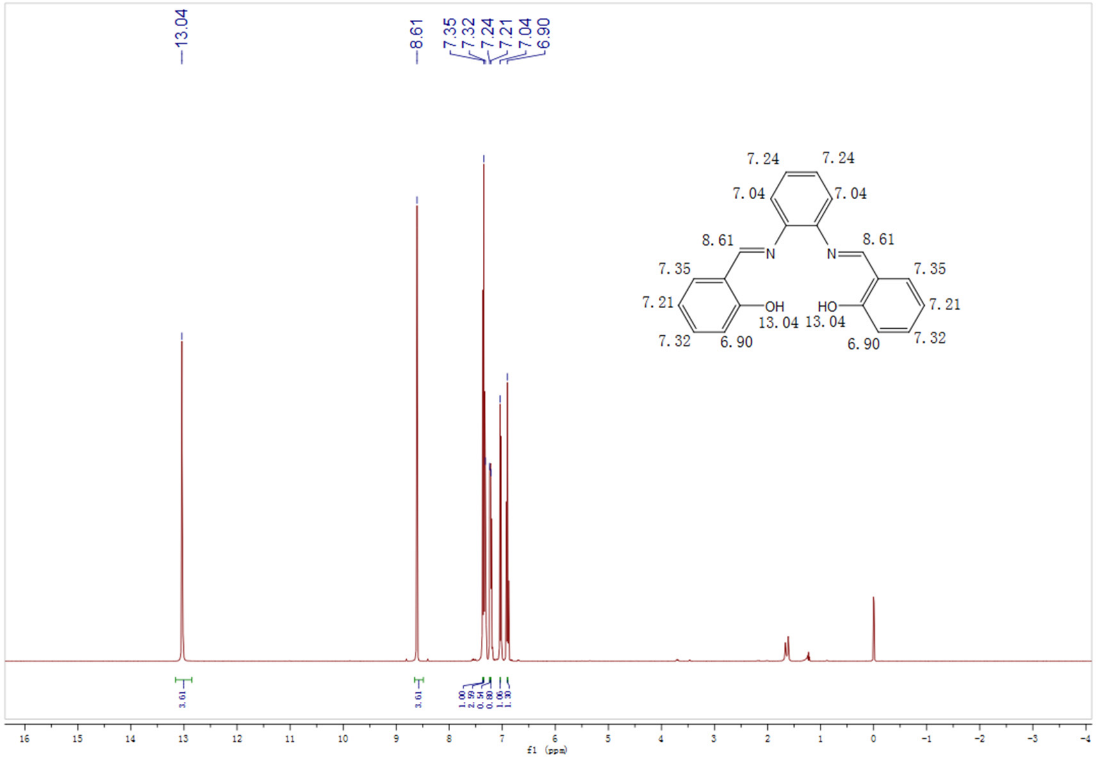

Figure S9. ^{1}H NMR ($CDCl_3$) of ligand salen-**1**.

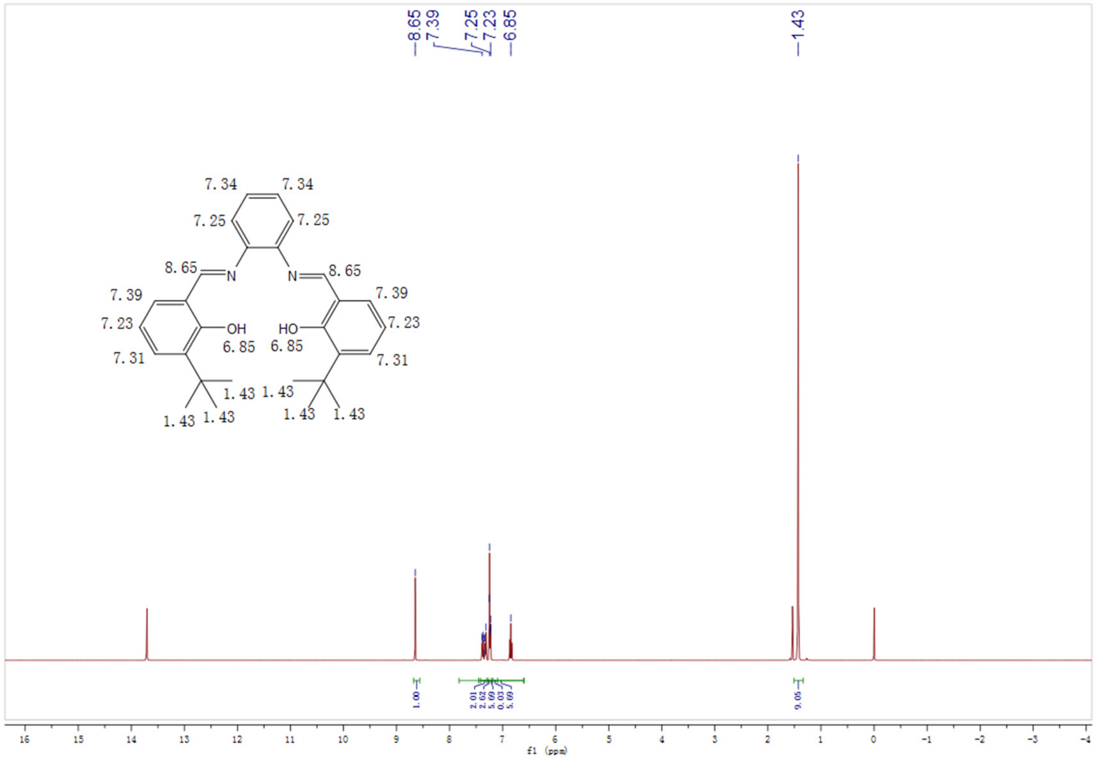

Figure S10. ^{1}H NMR ($CDCl_3$) of ligand salen-**2**.

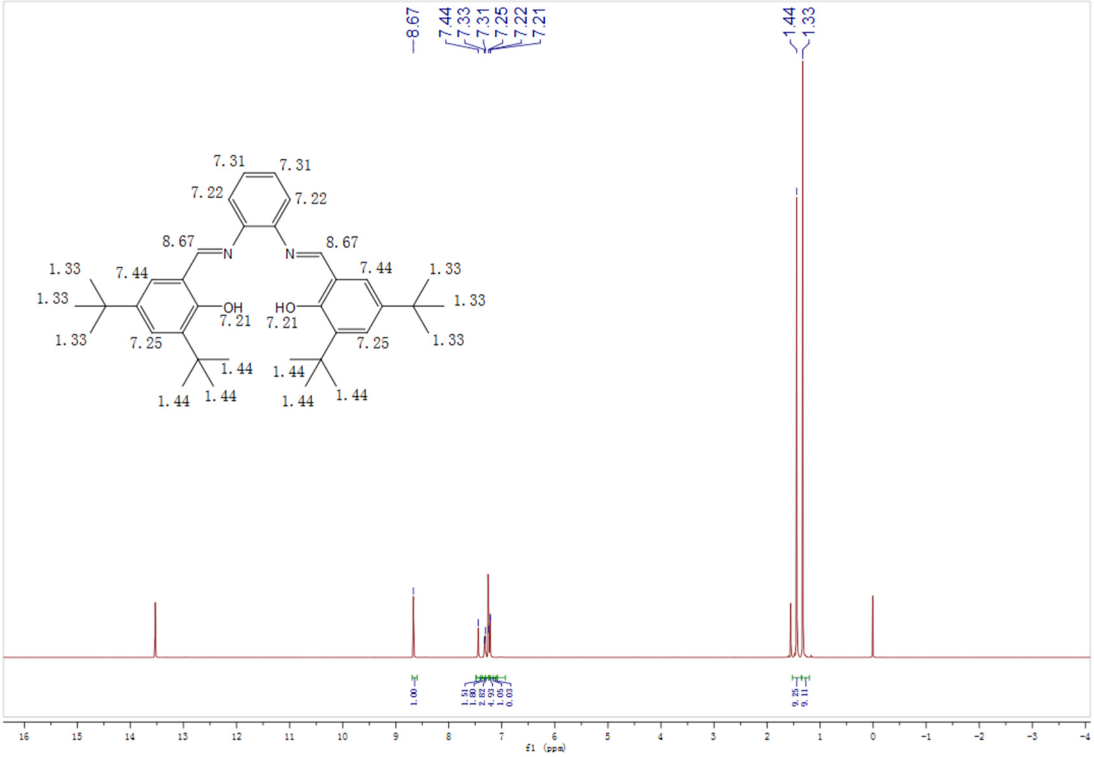

Figure S11. ^{1}H NMR ($CDCl_3$) of ligand salen-**3**.

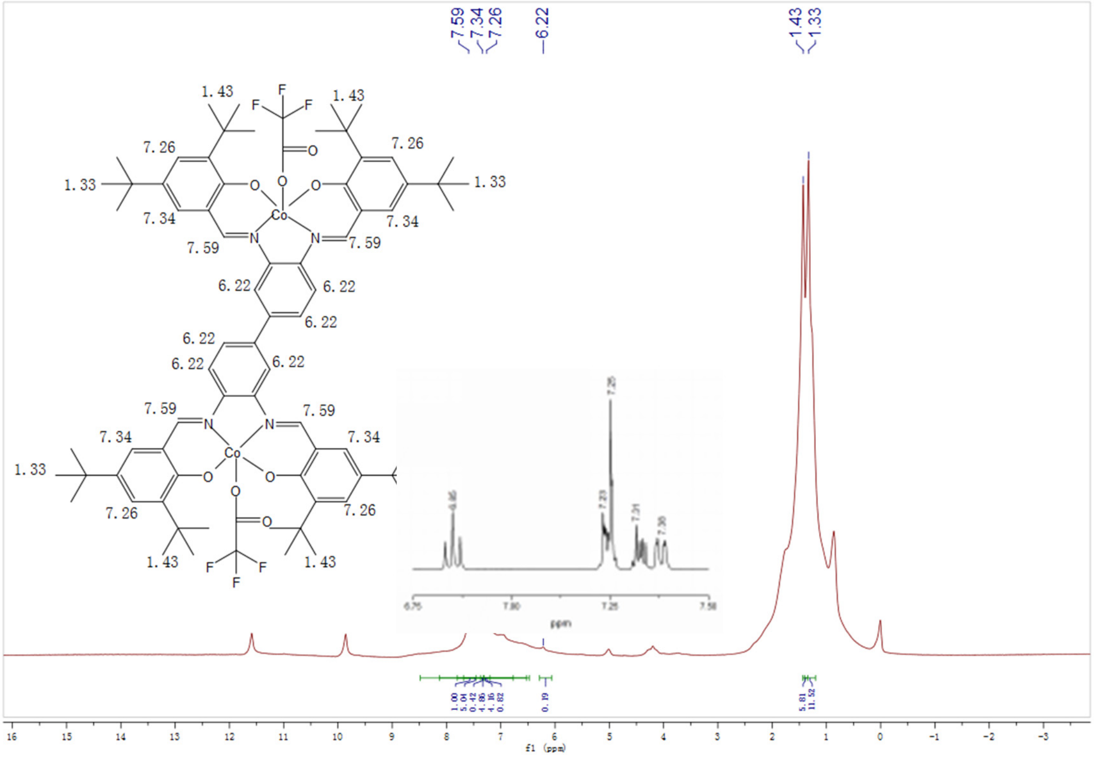

Figure S12. ^{1}H NMR ($CDCl_3$) of complex (salen-**4**)[Co(III)TFA]$_2$.

4. Characterization of the crude product PPC.

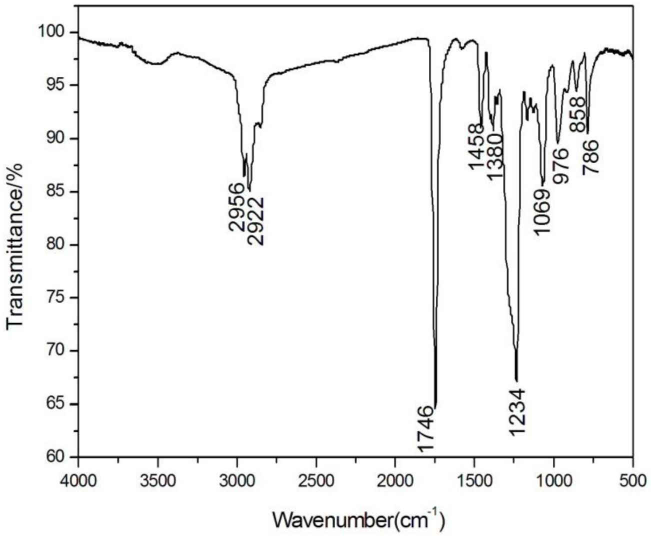

Figure S13. IR of the crude product PPC.

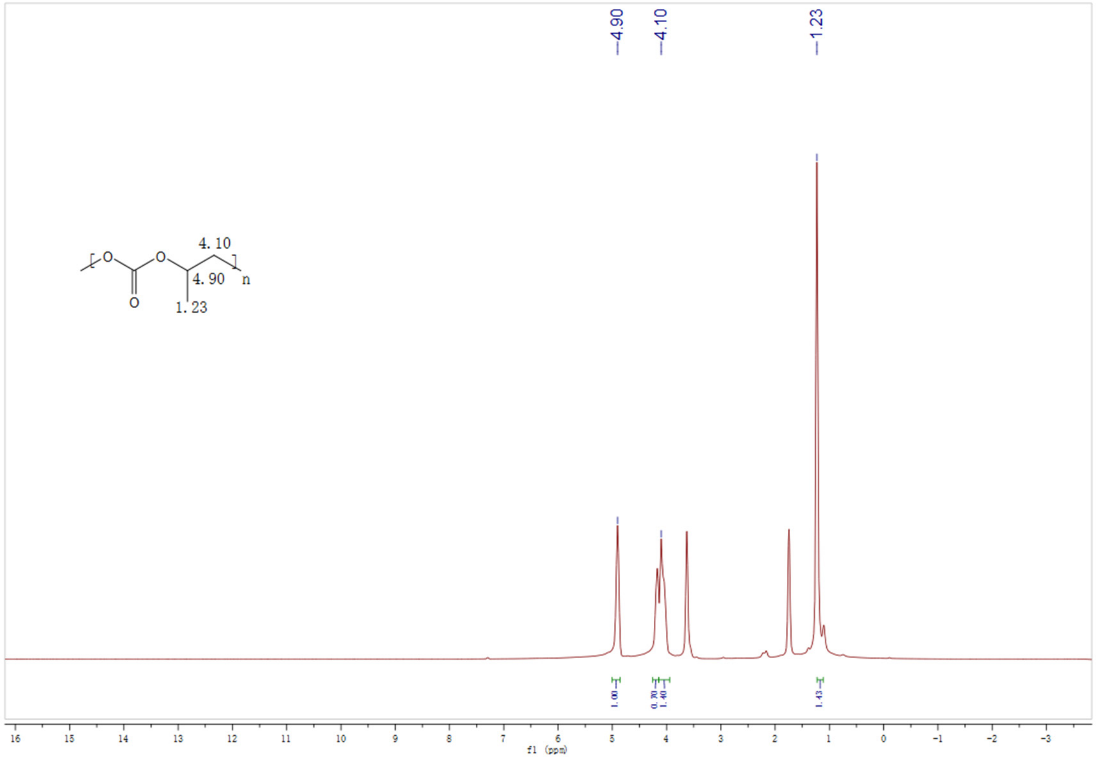

Figure S14. ^{1}H NMR ($CDCl_3$) of the crude product PPC.

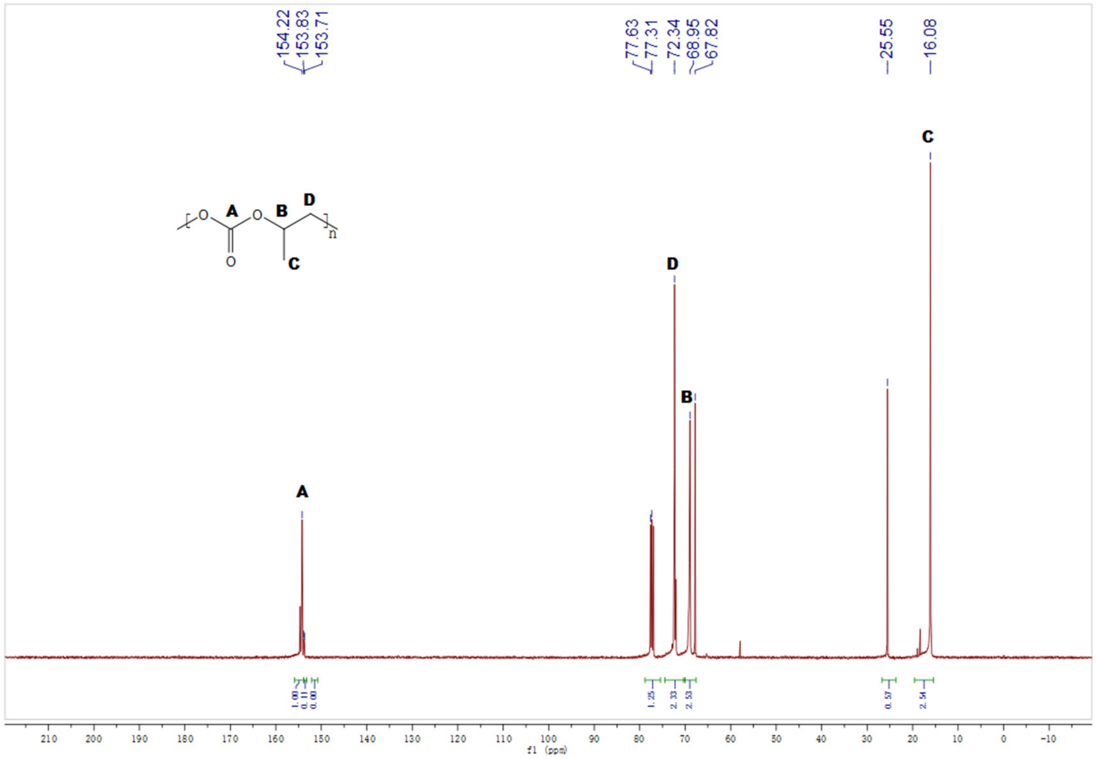

Figure S15. ^{13}C NMR ($CDCl_3$) of the crude product PPC.

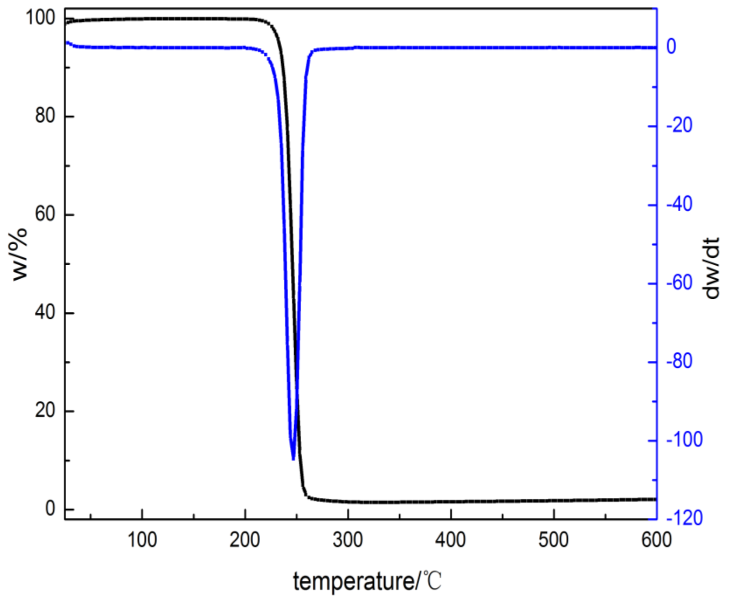

Figure S16. TG-DTG of the crude product PPC.

The Agilent PL-GPC 50 gel chromatograph and the American Jordi-Gel DVB organic phase gel chromatography column was used. Tetrahydrofuran as the mobile phase, adjusted the flow rate to 1 ml/min, the oven temperature was 40 ºC, the purified PPC was configured as 10 ppm concentration solution, after the baseline was stable, the relative molecular mass and related data were tested. (Fig. S17 and Table S1).

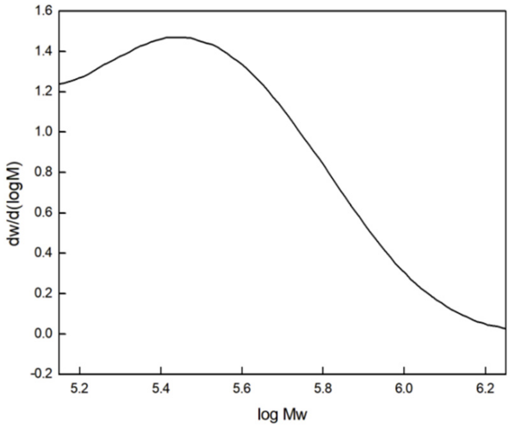

Figure S17. GPC of the crude product PPC.

Table S1. GPC of the crude product PPC.

	M_p (g/mol)	M_n (g/mol)	M_w (g/mol)	M_z (g/mol)	M_{z+1} (g/mol)	M_v (g/mol)	PDI
PPC	266286	293023	395054	552914	750654	375827	1.3482

5. The detailed quantum chemical theory calculation data.

Table S2. Bond lengths [Å] and angle [°] after optimizing for (salen-**4**)[Co(III)TFA]$_2$.

(salen-**4**)[Co(III)TFA]$_2$			
N(13)—Co(82)	1.90870	N(47)—Co(81)	1.90870
N(14)—Co(82)	1.91645	N(48)—Co(81)	1.90261
O(18)—Co(75)	1.88349	O(52)—Co(81)	1.86960
O(19)—Co(82)	1.88735	O(53)—Co(81)	1.86426
O(84)—Co(82)	1.87190	O(83)—Co(81)	1.85455
O(18)—Co(82)—O(19)	86.90	O(52)—Co(81)—O(53)	86.95
N(13)—Co(82)—N(14)	84.22	N(47)—Co(81)—N(48)	85.07
O(18)—Co(82)—N(14)	169.08	O(52)—Co(81)—N(48)	167.83
O(19)—Co(82)—N(13)	160.14	O(53)—Co(81)—N(47)	167.37
O(18)—Co(82)—N(13)	92.95	O(52)—Co(81)—N(47)	92.90
O(19)—Co(82)—N(14)	92.19	O(53)—Co(81)—N(48)	92.41
O(84)—Co(82)—N(14)	89.76	O(83)—Co(81)—N(48)	98.80
O(84)—Co(82)—N(13)	108.29	O(83)—Co(81)—N(47)	99.13
O(84)—Co(82)—O(18)	101.13	O(83)—Co(81)—O(52)	93.38
O(84)—Co(82)—O(19)	91.17	O(83)—Co(81)—O(53)	93.49

Table S3. NBO charges distribution of some atoms in complex (salen-**4**)[Co(III)X]$_2$.

(salen-**4**)[Co(III)Cl]$_2$		(salen-**4**)[Co(III)DNP]$_2$		(salen-**4**)[Co(III)TFA]$_2$	
Atom	Charge	Atom	Charge	Atom	Charge
Co(74)	0.544	Co(74)	0.740	Co(81)	0.754
N(43)	-0.413	N(43)	-0.448	N(47)	-0.488
N(44)	-0.461	N(44)	-0.447	N(48)	-0.446
O(48)	-0.567	O(48)	-0.565	O(52)	-0.574
O(49)	-0.556	O(49)	-0.565	O(53)	-0.576
Cl(76)	-0.315	O(76)	-0.558	O(83)	-0.489
Co(75)	0.545	Co(75)	0.737	Co(82)	0.754
N(13)	-0.489	N(13)	-0.462	N(13)	-0.446
N(14)	-0.489	N(14)	-0.441	N(14)	-0.488
O(18)	-0.564	O(18)	-0.574	O(18)	-0.574
O(19)	-0.563	O(19)	-0.566	O(19)	-0.576
Cl(77)	-0.276	O(77)	-0.587	O(84)	-0.489
